# Bone Mineral Density in Adult Patients with Type 1 Diabetes Mellitus Assessed by Both DXA and QCT

**DOI:** 10.1155/2023/8925956

**Published:** 2023-06-15

**Authors:** Eleftheria Barmpa, Spyridon Karamagkiolis, Stelios Tigas, Parthena Navrozidou, Marianna Vlychou, Ioannis Fezoulidis, Georgios N. Koukoulis, Alexandra Bargiota

**Affiliations:** ^1^Department of Endocrinology and Metabolic Diseases, University General Hospital of Larissa, Larissa, Greece; ^2^Department of Pathology, General Hospital of Larissa, Larissa, Greece; ^3^Department of Endocrinology, Medical School, University of Ioannina, Ioannina, Greece; ^4^Radiology Department Clinical and Laboratory Research, University General Hospital of Larissa, Larissa, Greece

## Abstract

**Purpose:**

Bone mineral density (BMD) was measured in uncomplicated young adult patients with type 1 diabetes mellitus (T1DM) and sex- and age-matched controls, using both dual X-ray absorptiometry (DXA) and quantitative computed tomography (QCT) to investigate their diagnostic ability in detecting abnormal values in these patients.

**Methods:**

118 patients with T1DM (65 females, mean age 30.12 ± 8.78 years) and 94 sex- and age-matched controls were studied. BMD was assessed in all participants by DXA and QCT at lumbar spine (LS). Biochemical markers of bone metabolism were also measured.

**Results:**

T1DM was associated with lower BMD at L1-L3 vertebrae measured by both DXA and QCT and lower bone turnover compared to sex- and age-matched controls. In T1DM subjects, QCT detected more patients with abnormal BMD values compared to DXA. BMI and HbA1c levels were the only determinants of BMD. Bone turnover markers were lower in patients with longer duration of diabetes.

**Conclusion:**

QCT provides a higher sensitivity compared to DXA in detecting abnormal BMD values in patients with uncomplicated T1DM. In these patients, the diabetes-related decreased BMD may be present early, before it is detected by DXA, the clinical gold standard for BMD measurements, and before the presence of any other diabetes complications, stressing the importance of an early intervention for fracture prevention.

## 1. Introduction

Current evidence suggests that type 1 diabetes mellitus (T1DM) affects bone health and is a risk factor for osteoporosis and osteoporotic fractures [1–4]. The overall and hip fracture risk is estimated to be threefold to sixfold higher compared to the general population, applies to both sexes and all ages, and is observed early on in life and maintained throughout [4–6]. Low bone mineral density (BMD) is considered to be one of the major risk factors for fractures at the spine and hip, and T1DM patients found to have reduced BMD, compared to nondiabetic individuals [5]. Even more, studies in children with T1DM found that a low for age BMD may be present early, after the diagnosis of the disease [7]. However, the increased fracture risk observed in patients with T1DM is much higher than expected based on the BMD indicating that BMD, routinely measured by dual X-ray absorptiometry (DXA), underestimates fracture risk in patients with T1DM especially in young adults [4].

DXA, the gold standard for measuring BMD, is simple to perform, not dependent on operator skills and experience, and is highly reproducible and with low radiation exposure [8], but it only measures BMD as an area density (two-dimensional method, 2D) without being able to differentiate between cortical and trabecular bone. Thus, it only measures the quantity of the bone and does not provide accurate information about bone integrity and microarchitecture [9].

Quantitative computed tomography (QCT) is a three-dimensional imaging technique that is also used for BMD measurements. Most commonly, it is applied to the spine, where typical lumbar vertebral elements are evaluated, but other skeletal sites such as the hip and the forearm can also be measured [10]. It provides multiplanar images from direct bone measurements, without the interference of the surrounding soft tissues, and thus, it allows direct measurements of bone's volume which is expressed directly as bone density [10]. Additionally, it has the ability to measure and distinguish trabecular from cortical bone and determine bone geometry, and therefore, QCT can identify vertebral fractures better than DXA [11, 12]. Moreover, QCT BMD measurements of the lumbar spine are independent of the subject's body size, in contrast to DXA measurements that are areal and are affected by the body size, and thus, QCT is considered a better tool for BMD evaluations in children and young adults, as well as for those with different statures and extreme BMIs [13–15].

Additional and more detailed information about bones' condition could be attained combining DXA and QCT for BMD measurements. Indeed, in studies in postmenopausal women with idiopathic osteoporosis, in patients with chronic obstructive pulmonary disease, in anorexia nervosa, and in patients who had bariatric surgery, the above-mentioned projecting errors of DXA were surpassed with the combined use of QCT for BMD measurements [16–19]. Even more, with the addition of QCT, low bone mass was detected early on allowing an earlier intervention and treatment when necessary.

T1DM is mainly diagnosed in childhood and in young adults. BMD changes can occur early in these patients, and considering that as much as 30% of adult bone mass is acquired during puberty [20], it is important to be able to diagnose and manage any bone changes right in the beginning in order to reduce fracture risk and its lifelong burden. However, BMD evaluations by DXA in children and young adults have its limitations due to their small body size, and the use of QCT might offer additional information. Data on the QCT measurements of bone density in lumbar spine are limited both in children [21] and in young adults [22] with T1DM. In patients with T1DM, mainly, peripheral QCT (pQCT) has been used in adolescent [23–25] and adult [26] populations to study bone geometry and BMD at peripheral sites. Studies where both methods were used for BMD measurements of the axial skeleton in this population are lacking. The aim of the current study was to examine BMD in uncomplicated young adult patients with T1DM and sex- and age-matched controls, using both DXA and QCT, and to assess the ability of these two methods in detecting abnormal values in these groups.

## 2. Materials and Methods

### 2.1. Patients

The study was designed as a single-center, case-control study. One hundred and eighteen (118) patients, 55% females, (65/118), with T1DM for more than 5 years, with an age range between 20 and 40 years old (mean age 30.12 ± 8.78 years), and without micro- or macrovascular complications were studied (T1DMG). Patients were recruited for the study from our outpatient clinics. Patients on corticosteroid treatment, with inflammatory diseases such as rheumatoid arthritis, malnutrition, renal failure (eGFR < 60 ml/min), or any other cause of secondary osteoporosis, were excluded from the study. Ninety-four (94) healthy subjects, matched for age, gender, and body mass index (BMI), were recruited from the university and hospital staff and formed the control group (CG). None of the female study participants was pregnant or postmenopausal. All participants were Caucasian. Signed informed consent forms were obtained from all individual participants included in the study. The study was approved by the hospital's ethical committee.

### 2.2. Bone Measurements

#### 2.2.1. DXA Examination

A Hologic Discovery QDR Series Densitometer (Hologic Inc., Bedford, MA) was used in our study to measure BMD in all participants at lumbar spine (LS). The device was daily calibrated for quality control, and the coefficient of variation (CV) for the spine phantom was 1.08%. Absolute values of BMD (in g/cm^2^) and, due to the young age of the participants, the *Z*-score were measured. Total LS BMD (L1-L4) was calculated, and for ROI selection, we compared L1, L2, and L3 vertebrae separately with the corresponding measurements from QCT. Based on the definition of the International Society for Clinical Densitometry (ISCD), a *Z*-score of -2.0 or lower was defined as “below the expected range for age”, and a *Z*-score above -2.0 was “within the expected range for age” [8]. Areas of sclerosis or osteophytes were excluded from the analysis.

#### 2.2.2. QCT Examination

A Toshiba (Tokyo, Europe) Aquilion 16-slice-computed tomography device and a solid QCT phantom (Mindways Software Inc., Austin, TX, USA) were used for the QCT measurements. Scan parameters were 120 kV, 100 mAs, and 1 mm slice thickness. Daily calibrations for quality control were performed, and the CV for the phantom was 3.8 ± 2.2%. For 2D single-slice QCT, generally, three lumbar vertebrae are scanned, usually the L1-L3. Initially, a lateral scan projection radiograph is obtained, and the slices to be performed are identified in the midplane of each selected vertebra and parallel to the endplates; the slice width is 10 mm, and the low-density area in the posterior aspect of the body marks the entry of the basivertebral vein and confirms the section to be in the midplane of the vertebra [10]. In our study, the phantom was placed on the midline in the thoracolumbar region, and the images taken were examined for any loss of vertebral height or wedge deformity, compatible with osteoporotic fracture, which together with any vertebral osseous lesions were excluded from the 10 mm-thick nonangled reconstructions that were made through the center of each L1-L3 vertebra.

For trabecular BMD measurements a software package, QCT PRO 4.2.3, was used in the present study, and an oval region of interest (ROI) was placed in the trabecular bone at the anterior part of three vertebral bodies (L1-L3), according to ISCD for single-slice QCT [8], excluding areas of sclerosis and the area of the basivertebral vein and the vertebral cortex. Based on the recent (revised in 2018) ACR guidelines for premenopausal women, men younger than 50, and children, QCT reports should include BMD values and *Z*-scores. *Z*-scores above -2.0 are within the expected range, and *Z*-scores of -2.0 or lower are considered to be below the expected range for age [27].

In all subjects, the DXA and QCT measurements were performed on the same day.

### 2.3. Anthropometric Characteristics and Biochemical Assays

Body weight was measured to the nearest 0.1 kg with patient in light clothing and shoes removed. Height was measured using a wall-mounted stadiometer and recorded to the nearest 0.1 cm. BMI was calculated as kg/m^2^.

Blood samples were collected at fasting conditions for beta-crosslaps (*β*-crosslaps) as a measure of bone resorption and total procollagen type 1 amino-terminal propeptide (TP1NP) as a measure of bone formation and stored at −80°C until analysis. Both markers were measured by using the enzyme-linked immunosorbent assay (Elisa) (Elecsys 1010/2010/MODULAR ANALYTICS E170). Glycosylated hemoglobin (HbA1c) was measured by capillary electrophoresis (Capillarys 2 Flex Piercing, Sebia, Lisses, France) at our hospital's laboratory of biochemistry.

### 2.4. Statistical Analysis

Categorical variables are reported as absolute numbers; continuous variables were reported as means ± standard deviations (mean ± SD). Comparison of frequencies of categorical variables between two groups was performed by the *χ*^2^ test or Fisher's exact test in case of small frequencies. Comparison of continuous variables between two groups was performed using two-sample independent samples *t*-test, when data were normally distributed, and by Mann–Whitney *U*-test, in other cases. Two tailed *p* values <0.05 were considered significant. Pearson's correlation analyses were used to assess the univariate relationship between BMD and risk factors. A value of *p* < 0.05 was considered as statistically significant. All data analysis was conducted using the SPSS version 25 (IBM SPSS Statistics for Windows, Armonk, NY, USA).

## 3. Results

Anthropometric characteristics, HbA1c values, and gender distribution of T1DMG and CG subjects are presented in Table 1. There was no difference in age, gender, and BMI between the two groups. In T1DMG, mean age of diabetes diagnosis was 19.17 ± 9.80 years, mean disease duration was 16.16 ± 9.56 years and mean Hb1Ac was 7.85 ± 1.43%.

Results of BMD measurements by DXA are shown in Table 2. Absolute values of BMD (g/cm^2^) measured by DXA were significantly lower at total LS (*p* = 0.018) and at L1 (*p* = 0.004), L2 (*p* = 0.019), and L3 (*p* = 0.020) vertebrae, in T1DMG compared to CG. Males with T1DM had significantly lower BMD values at above sites compared to sex-matched controls, in contrast to females which these reduced BMD values did not reach statistical significance.

Results of BMD measurements by QCT are shown in Table 3. QCT BMD measurements in T1DMG were significantly lower at all examined vertebrae (L1, L2, and L3) compared to CG (*p* = 0.011, *p* = 0.017, and *p* = 0.002, respectively). Males with T1DM had significantly lower BMD values compared to controls in all vertebrae (L1: *p* = 0.001, L2: *p* = 0.001, and L3: *p* ≤ 0.001). In contrast, in females, no difference was observed in BMD measurements between the two groups at all vertebrae (L1: *p* = 0.848, L2: *p* = 0.745, and L3: *p* = 0.504).

On a per-patient basis, Table 4 shows the ability of DXA and QCT to detect abnormal values in the LS of all patients and both sexes separately. In T1DMG, QCT detected overall more patients with BMD values below the expected range for age (*Z*-score, below -2.0) compared to DXA (*Z*-score, below -2.0). In particular, QCT detected 11 patients (9.2%) at L1, 9 patients (7.6%) at L2, and 10 patients (8.4%) at L3 vertebra *Z*-score below -2.0. DXA detected 3 patients (2.5%) at L1, 2 patients (1.7%) at L2, and 4 patients (3.4%) at L3 vertebra with *Z*-score below -2.0. In the CG, all subjects had *Z*-scores above -2.0 at all sites measured by both DXA and QCT.

### 3.1. Bone Markers

Results of bone markers are shown in Table 1. *β*-Crosslap values were significantly lower in T1DMG compared to CG (*p* = 0.001), and this difference occurred in both sexes (males: *p* = 0.028 and females: *p* = 0.005). TP1NP values in T1DMG were similar compared to CG (*p* = 0.178). TPINP values were also similar for males and females with T1DM compared to controls (males: *p* = 0.589; females: *p* = 0.392).

### 3.2. Determinants of BMD in Patients with T1DM

*Ι*n T1DMG, there was a statistically significant positive correlation between BMI and *Z*-scores, measured by DXA at LS (*r* = 0.236, *p* = 0.010), as well as between BMI and BMD measured by QCT (L1: *r* = 0.321, *p* = 0.024; L2: *r* = 0.041, *p* = 0.031; L3: *r* = 0.041, *p* = 0.035). HbA1c levels in T1DMG were negatively correlated with QCT BMD measurements at all vertebrae (L1: *r* = −0.262, *p* = 0.011; L2: *r* = −0.253, *p* = 0.014; L3: *r* = −0.221, *p* = 0.034). No correlation was found between HbA1c and DXA-measured BMD. No statistically significant correlation was found between age, age at diabetes diagnosis, and duration of diabetes with either DXA or QCT measurements.

In T1DMG, both *β*-crosslaps and TP1NP were significantly and negatively correlated with the duration of diabetes (*r* = −0.378, *p* ≤ 0.001 and *r* = −0.322, *p* = 0,003, respectively). Age at diabetes diagnosis was significantly negatively correlated with TP1NP (*r* = −0.223, *p* = 0.040). Negative correlation was found between *β*-crosslaps and total BMD, measured by DXA in T1DMG, at LS (*r* = −0.302, *p* = 0.012). In addition, negative correlation was found between *β*-crosslaps and L1, L2, and L3 measured by QCT (L1: *r* = −0.327, *p* = 0.023; L2: *r* = −0.299, *p* = 0.036; L3: *r* = −0.411, *p* = 0.029). No correlation was found between both markers and either BMI or HbA1c.

## 4. Discussion

To our knowledge, this is the first study assessing BMD using both DXA and QCT in patients with T1DM. Our data show that patients with T1DM have a lower BMD at LS, measured by both DXA and QCT and lower *β*-crosslaps in comparison to sex- and age-matched controls. In T1DM subjects, QCT detected more patients with abnormal BMD values, a major determinant of the fracture risk in later life, compared to DXA. BMI was the only determinant of BMD, measured by both DXA and QCT, and HbA1c was a determinant only for QCT measured BMD. Bone turnover markers were lower in patients with longer duration of diabetes.

Our findings on DXA-measured BMD are in line with the findings of two meta-analysis that also reported a modestly lower LS BMD compared to controls when adjusted by age, sex, and DXA techniques [4, 28]. In contrast, in another meta-analysis by Pan et al. [29], pooled differences in BMD at LS were not different between patients with T1DM and controls. In this meta-analysis, though, a mixed population with studies in children, adolescents, and adults with T1DM was included and is possible, the age differences of the subjects, the variable pubertal stages, and the different DXA instruments used, might explain the different findings from our study, where uncomplicated young adults with T1DM were studied using the same DXA instrument.

In our study, QCT BMD measurements of LS were also lower in patients with T1DM compared to controls. Moreover, QCT detected more patients with abnormal BMD values of the L1-L3 vertebrae compared to DXA. DXA measurement technique has methodological problems and disadvantages. DXA is a 2D technique, and the given BMD includes measurements of the cortical and the trabecular bone of the vertebral body and the posterior elements and is dependent on bone size. Thus, the sensitivity of the method is decreased, especially when assessment of the small changes occurring in the metabolically active trabecular bone is needed [30]. Different attempts have been proposed in the literature to eliminate the DXA methodological disadvantages, either by calculating apparent volumetric BMD [31] or by using a DXA-based 3D-modelling of the spine [32], but such approaches have not been included in our initial study design. In contrast to DXA, QCT estimates a portion of the trabecular bone inside the vertebral body. Vertebral BMD using QCT was previously studied only in 48 children and adolescences, with uncomplicated T1DM but a broad age range (5.2 to 19.6 years), and found that a calculated index of cortical bone was slightly but significantly lower in patients compared to controls, while trabecular BMD was not different [21]. More widely in T1DM, pQCT has been used in studies in children, adolescents [23–25, 33], and less often in adults [26, 34], to assess cortical and trabecular bone characteristics of the distal radius and tibia in an effort to determine the etiology of the observed increased fracture risk. In these studies, all subjects with T1DM, children, adolescents, and adults found to have a lower BMD compared to controls. However, bone at these peripheral anatomical sites is primarily cortical, and their BMD measurements by pQCT are predominantly influenced by cortical bone. On the contrary, the axial skeleton is composed mostly of trabecular bone that has a significantly higher metabolic turnover than the cortical and is very susceptible to early and severe manifestations of BMD changes. QCT is considered to be more sensitive in monitoring disease and treatment-related BMD changes [10, 35]. Additionally, QCT is less susceptible to degenerative changes of the spine and joints as well as to soft tissue calcifications [35]. These confounding factors which influence the BMD measurement of both methods were minimized in our study where adult healthy controls and patients of relatively young age and uncomplicated T1DM were included.

The ability of both DXA and QCT to assess BMD changes has been studied outside diabetes in different diseases. A study in postmenopausal women showed that QCT at LS had a greater diagnostic sensitivity than DXA to detect osteoporosis [16], and in another one, in male patients with chronic obstructive pulmonary disease, QCT BMD measurements found to be more valuable in estimating bone loss of the LS, compared to DXA [17]. Also, QCT found to be better than DXA in estimating bone loss of the LS among patients with spinal cord injury [36]. The superiority of QCT, compared to DXA, in detecting abnormal BMD in adult patients with T1DM was also found in our study.

In our study, a BMD analysis by gender showed that a significantly lower BMD, measured both by DXA and QCT, was observed only in male patients with T1DM but not in females, compared to controls. In the literature, reports have been variable about sex differences in bone health in T1DM. In agreement with our findings, a study dealing with bone mass and structure in adolescences aged 12-17 years showed that boys with T1DM were more affected than girls [24]. Moreover, studies in adults with T1DM have shown that the reduction in BMD was greater in men than that in women [37, 38]. Another study though, which included patients aged 6 to 20 years, found that girls with T1DM had significantly lower LS and total body BMC than control girls, whereas no such difference was observed in boys [39]. The broad age range of the subjects included in this study implies different stages of their bone maturation which might have been responsible for these findings. In our study, males and females with T1DM had similar age, BMI, age of diabetes diagnosis, diabetes duration, and HbA1c. The differences in BMD between sexes may reflect a different sex hormone impact on bone in the two genders. In premenopausal females, estrogen adequacy could act protectively on the skeleton against the detrimental effect of diabetes [40]. On the other hand, in males with T1DM, the described decline in the gonadal function, attributed defects caused by diabetes either at the hypothalamic–pituitary–gonadal axis or at testicular level, might also contribute to a negative effect on bone health [41, 42].

Up to date, there is no agreement regarding the parameters that determine BMD in patients with T1DM. Data in the literature are inconclusive, and results are variable. Studies have shown that T1DM is associated with a low bone turnover state, founding that CTX and osteocalcin were consistently lower in patients compared to controls, indicating that both bone resorption and formation are reduced [43, 44]. In a meta-analysis by Hygum et al., TP1NP was consistently lower in patients with T1DM compared with controls, but this difference did not reach significance [44]. In our study, we also found a reduced bone resorption as indicated by the lower *β*-crosslaps in patients with T1DM compared to controls, but we found no difference in TP1NP between patients and controls. In patients with T1DM, BMD also found to be correlated with glycemic control in a few studies [45, 46]. In our study, HbA1c was not correlated with the LS BMD measured by DXA. Also previously in a meta-analysis, in patients with T1DM, no association was found between HbA1c and BMD measured by DXA at LS when controlling for age, sex, and DXA instrument [28]. In our study though, HbA1c was negatively correlated with QCT-measured BMD at L1, L2, and L3 in patients with T1DM, which might be explained by the superiority of QCT compared to DXA in measuring changes occurring at trabecular bone which is the predominant at vertebrae. However, a single HbA1c measurement may not adequately reflect the effect of long-term bone glycemic exposure and thus might not be able to explain the detrimental effects of chronic hyperglycemia on bone. In studies that included patients with microvascular diabetic complications, an indicator of chronic poor diabetic control, an association between the presence of complications and the presence, and/or progression of a decreased BMD were reported [4]. In our study, we excluded patients with micro- and macrovascular diabetic complications, but we still did not find a correlation between poor glycemic control and reduced BMD measured by DXA. The age of T1DM diagnosis may be crucial for the acquisition of bone mass, and a diagnosis at an early age can be a risk factor for smaller bone size [24]. But as previously shown in the literature [28], we also did not find any correlation between BMD, measured by both methods, and duration of diabetes. BMI is generally considered to play a protective role on bones according to our findings and those of some previous studies in the literature [4].

Our study has limitations. One limitation is that no volumetric QCT measurements were obtained, because of the CT software that was available. However, the single-slice QCT BMD measurements that were obtained in our study are of clinical significance and importance. Also, from a DXA point of view, for patient's classification except the spine measurements, hip sites are also needed which are not presented here. We used the L1-L3 for DXA measurements because the hip region is a late responder to bone loss; vertebral bodies are more sensitive to bone mineral changes compared to any other skeletal region as they are rich in trabecular bone, and thus, they are the preferred area for early change detection and QCT comparison. Another limitation is that we enrolled participants at a single urban academic center, which may not be the representative of other sites. Moreover, a larger number of patients may be needed to specify possible determinants of BMD measurements by both methods. The strengths of our study are that all our patients were young adults with uncomplicated T1DM of relatively long duration and well matched with CG. Thus, we avoided potential interference of puberty and diabetic complications on bone accrual and density. Moreover, none of the women included in the study was peri- or postmenopausal.

## 5. Conclusions

In conclusion, our findings indicate that QCT has a better diagnostic ability in detecting lower BMD values compared to DXA in young uncomplicated patients with T1DM. These patients compared to matched controls have a lower BMD, measured by both DXA and QCT, associated with a lower bone turnover and BMI. Glycemic control was a determinant only for QCT-measured BMD. Our data indicate that DXA, the standard and widely available technique used routinely to measure BMD, underestimates the reduced BMD, and there are more patients with T1DM than those detected by DXA who may be at risk of a fracture, even before the presence of any other diabetes-related complications. Assessment and management of reduced BMD in young patients with T1DM are complex and challenging, and fracture prediction tools are not validated. As these patients may present with BMD changes early after the diabetes diagnosis, our study results stress the need of a prompt therapeutic intervention, with either lifestyle changes or pharmacologic therapy initiation, for reducing fracture risk and the lifelong disease burden. Moreover, as the two techniques measure different bone characteristics, they may be used as supplementary at an early stage in T1DM patients to detect early bone changes.

## Figures and Tables

**Table 1:** General characteristics of the participants in the study and gender distribution.

Variable	All			Males			Females		
	T1DM (*n* = 118)	CG (*n* = 94)	*p*	T1DM (*n* = 53)	CG (*n* = 42)	*p*	T1DM (*n* = 65)	CG (*n* = 52)	*p*
Age (years)	30.12 ± 8.78	29.81 ± 7.60	0.311	29.19 ± 7.74	28.73 ± 6.61	0.867	31.05 ± 8.19	30.89 ± 7.88	0.101
Height (cm)	170 ± 10	171 ± 9	0.428	178 ± 7	180 ± 6	0.357	163 ± 6	164 ± 5	0.413
Weight (kg)	74.65 ± 15.29	78.67 ± 16.08	0.070	82.16 ± 13.48	87.82 ± 14.23	0.054	68.52 ± 13.96	71.21 ± 13.52	0.304
BMI (kg/m^2^)	25.74 ± 4.78	26.59 ± 4.73	0.164	25.64 ± 3.87	27.00 ± 4.05	0.102	25.39 ± 4.24	26.26 ± 5.22	0.650
Age of diagnosis (years)	19.17 ± 9.80	—	—	19.62 ± 10.45	—	—	18.80 ± 9.30	—	—
Disease duration (years)	16.16 ± 9.56	—	—	15.72 ± 9.40	—	—	16.52 ± 9.76	—	—
HbA1c (%)	7.85 ± 1.43	5.12 ± 0.26	≤0.001	7.80 ± 1.25	5.13 ± 0.27	≤0.001	7.88 ± 1.56	5.11 ± 0.26	≤0.001
*β*-Crosslaps (pg/ml)	329.16 ± 199.87	442.81 ± 220.66	0.001	302.79 ± 130.46	481.55 ± 119.45	0.028	262.94 ± 139.89	368.81 ± 133.35	0.005
TP1NP (pg/ml)	51.98 ± 33.25	53.12 ± 24.43	0.178	62.01 ± 39.89	66.30 ± 34.72	0.589	43.07 ± 22.96	46.53 ± 13.73	0.392

Data are expressed as mean ± SD. *n*: number of patients; BMI: body mass index.

**Table 2:** LS measurements by DXA in the study population and gender distribution.

Variable		All			Males			Females		
		T1DM (*n* = 118)	CG (*n* = 94)	*p*	T1DM (*n* = 53)	CG (*n* = 42)	*p*	T1DM (*n* = 65)	CG (*n* = 52)	*p*
LS										
Total	BMD (g/cm2)	1.06 ± 0.13	1.10 ± 0.14	0.018	1.05 ± 0.13	1.12 ± 0.15	0.004	1.06 ± 0.13	1.07 ± 0.13	0.654
	*Z*-score	0.11 ± 1.25	0.65 ± 1.22	0.001	−0.20 ± 1.24	0.64 ± 1.41	0.002	0.36 ± 1.20	0.66 ± 1.04	0.285
L1	BMD (g/cm^2^)	1.01 ± 0.13	1.06 ± 0.14	0.004	1.01 ± 0, 12	1, 09 ± 0, 15	0.006	1.00 ± 0.13	1.03 ± 0.12	0.710
	*Z*-score	0.10 ± 1.23	0.65 ± 1.23	0.001	−0.29 ± 1.18	0.49 ± 1.4	0.003	0.42 ± 1.17	0.81 ± 1.01	0.241
L2	BMD (g/cm^2^)	1.08 ± 0.13	1.11 ± 0.14	0.019	1.07 ± 0.12	1.14 ± 0.15	0.014	1.08 ± 0.14	1.09 ± 0.12	0.355
	*Z*-score	0.43 ± 1.27	0.90 ± 1.25	0.006	0.04 ± 1.18	0.83 ± 1.45	0.003	0.75 ± 1.26	0.97 ± 1.03	0.303
L3	BMD (g/cm^2^)	1.08 ± 0.14	1.12 ± 0.15	0.020	1.07 ± 0.13	1.15 ± 0.16	0.010	1.09 ± 0.13	1.10 ± 0.14	0.987
	*Z*-score	0.15 ± 1.30	0.63 ± 1.33	0.007	−0.10 ± 1.28	0.72 ± 1.50	0.004	0.35 ± 1.29	0.55 ± 1.16	0.469

Data are expressed as mean ± SD; *n*: number of patients; BMD: bone mineral density; LS: lumbar spine; L1: lumbar vertebrae 1; L2: lumbar vertebrae 2; L3: lumbar vertebrae 3.

**Table 3:** QCT-measured BMD and gender distribution.

Variable QCT		All			Males			Females		
		T1DM (*n* = 118)	CG (*n* = 94)	*p*	T1DM (*n* = 53)	CG (*n* = 42)	*p*	T1DM (*n* = 65)	CG (*n* = 52)	*p*
L1	BMD (g/cm3)	180.82 ± 34.90	192.83 ± 32.15	0.011	171.35 ± 39.86	199.53 ± 36.99	0.001	187.55 ± 29.98	188.54 ± 28.30	0.848
	*Z*-score	0.0 ± 1.02	0.61 ± 1.12	≤0.001	−0.43 ± 1.11	0.52 ± 1.31	≤0.001	0.33 ± 1.57	0.74 ± 1.12	0.199
L2	BMD (g/cm3)	178.88 ± 34.07	189.64 ± 29.47	0.017	169.21 ± 38.39	195.37 ± 32.88	0.001	185.12 ± 25.92	186.76 ± 28.00	0.745
	*Z*-score	0.19 ± 1.34	0.79 ± 1.18	0.005	−0.13 ± 1.24	0.78 ± 1.39	0.001	0.63 ± 1.15	0.89 ± 1.15	0.445
L3	BMD (g/cm3)	175.30 ± 34.05	189.11 ± 29.53	0.002	166.67 ± 37.53	193.19 ± 31.97	≤0.001	182.34 ± 29.39	185.90 ± 27.34	0.504
	*Z*-score	−0.10 ± 1.21	0.57 ± 1.41	0.005	−0.34 ± 1.15	0.61 ± 1.39	0.002	0.24 ± 1.35	0.42 ± 1.02	0.432

Data are expressed as mean ± SD; *n*: number of patients; BMD: bone mineral density. L1: lumbar vertebrae 1; L2: lumbar vertebrae 2; L3: lumbar vertebrae 3.

**Table 4:** DXA and QCT ability to detect BMD values below the expected range for age, per vertebra level, and gender distribution.

*Z*-score ≤ -2.0	All		Males		Females	
	DXA (*n* = 9)	QCT (*n* = 30)	DXA (*n* = 5)	QCT (*n* = 20)	DXA (*n* = 4)	QCT (*n* = 10)
L1	3	11	2	7	1	4
L2	2	9	1	6	1	3
L3	4	10	2	7	2	3

*n*: number of patients; L1: lumbar vertebrae 1; L2: lumbar vertebrae 2; L3: lumbar vertebrae 3.

## Data Availability

Raw data were generated at the Department of Endocrinology and Metabolic Diseases, University Hospital of Larissa, Greece. Derived data supporting the findings of this study are available from the corresponding author (E.B.) on request.
